# Insights into the mitochondrial DNA genetic diversity and affinities of village chickens from the Sulu Archipelago, Philippines

**DOI:** 10.1098/rsos.250633

**Published:** 2025-09-10

**Authors:** Michael James B. Herrera, Fairuz B. Bangahan, Mark Laurence D. Garcia, Michelle S. Eusebio, John Meldwin D. Cuales, Richard N. Muallil, Altan I. Ishmael, Raquel O. Rubio, Rolly C. Urriza, Jazelyn M. Salvador, Jae Joseph Russell B. Rodriguez, Maria Corazon A. De Ungria

**Affiliations:** ^1^ School of Archaeology, University of the Philippines Diliman, Quezon City, Metro Manila, Philippines; ^2^ DNA Analysis Laboratory, Natural Sciences Research Institute, University of the Philippines Diliman, Quezon City, Metro Manila, Philippines; ^3^ Office of Continuing Education and Extension Services, Mindanao State University - Tawi-Tawi College of Technology and Oceanography, Bongao, Tawi-Tawi, BARMM, Philippines; ^4^ Sama Studies Center, Mindanao State University - Tawi-Tawi College of Technology and Oceanography, Bongao, Tawi-Tawi, BARMM, Philippines; ^5^ Biological Research and Services Laboratory, Natural Sciences Research Institute, University of the Philippines Diliman, Quezon City, Metro Manila, Philippines; ^6^ Ornithology Section, Zoology Division, National Museum of Natural History, National Museum of the Philippines, Manila, Metro Manila, Philippines; ^7^ Department of Archaeogenetics, Max Planck Institute for Evolutionary Anthropology, Leipzig, Germany

**Keywords:** Philippines, Sulu Archipelago, chicken, mitochondrial DNA, human migration, Island Southeast Asia

## Abstract

The Sulu archipelago in the Philippines witnessed significant human migration from prehistoric to subrecent periods, resulting in a tapestry of cultures, languages, and genes. However, many details remain unclear. Archaeological evidence shows that human populations traversing within and between regions in Island Southeast Asia (ISEA) and the Pacific brought a variety of domestic animals, including dogs, pigs, chickens, and commensal Pacific rats. Recent insights into the ancient movements of these species suggest that they have different origins and arrival times. Here, we leveraged chickens as a tool to investigate human interactions in the Sulu archipelago. We sequenced a 764-base pair fragment of the mitochondrial DNA control region to examine the genetic diversity and structure of 254 village chickens from the Sulu archipelago. Combined with comparable datasets from other islands of ISEA and the Pacific, our data reveal a higher maternal genetic affinity of chickens from the Sulu Archipelago with those from adjacent Indonesian islands than with chickens from the rest of the Philippines. This observation points to long-standing regional interactions between the Sulu archipelago and Indonesia. Furthermore, core haplogroup D lineages, which characterise ancient Pacific chickens, are not found in the Sulu archipelago, but are present elsewhere in the Philippines.

## 1. Introduction

The last prehistoric migration of modern humans involved ocean voyages from Island Southeast Asia (ISEA) to the remote islands of the Pacific. The predominant model for this diaspora suggests that around 4200−4000 years before present (yBP), Austronesian-speaking people left Taiwan for the Philippines and areas further south, eventually occupying most of ISEA [1]. When people reached Near Oceania *ca* 3300 yBP, their culture became identifiable by its distinctive ‘Lapita’ pottery type. The continued colonisation of Remote Oceania soon followed [2]. Surviving these increasingly longer sea voyages and subsequent colonisation of depauperate island environments requires specific adaptive strategies, including the deliberate translocation of plants and animals [3,4]. Among the animals transported by early Polynesians, three were domesticated: dog (*Canis* spp.), pig (*Sus scrofa*), and chicken (*Gallus gallus*), which appear to have different origins in Mainland Southeast Asia (MSEA) [5–8]. The transport of these domesticates to Near and Remote Oceania, along with rats (*Rattus* spp.) [4,9], makes them highly informative as proxies for understanding patterns of prehistoric human migration in the region. Combining recent genetic studies with zooarchaeological research has highlighted the complex and potentially independent translocation histories of these animals in ISEA and the Pacific [10,11]. Dogs [5,12–14] and pigs [15,16] showed patterns consistent with a translocation route from MSEA through the Malay Peninsula and into ISEA, where they are ubiquitous across most islands and archipelagos, except for the Philippines. From eastern Indonesia, pigs and dogs were subsequently transported through Near and Remote Oceania. However, studies on the Pacific rat (*Rattus exulans*) [9] and chickens [8] suggest separate homelands within ISEA. Notably, of the four bioproxies, only chickens have a modern distribution that is still relatively understudied, especially in ISEA.

Most modern genetic studies on chickens have focused on MSEA and the Indian subcontinent as putative separate domestication centres [6,17]. Although relevant to understanding the origin of Pacific chickens, these studies have provided some insights into the translocation history of chickens through ISEA and Oceania. Archaeological records from the Bismarck Archipelago and Near Oceania have shown that chickens were associated with the Lapita Culture from at least 3000 yBP [18,19]. However, archaeological remains of chickens are extremely rare in ISEA. Consequently, little is known about the origins and timing of their introduction or translocation routes across ISEA.

Understanding the genetic diversity of chickens across ISEA is essential for reconstructing migration models from MSEA to the Pacific. Of the 13 divergent chicken mitochondrial DNA (mtDNA) haplogroups (A–I defined by sequence variants in the control region (CR); W–Z defined by the sequence variants in the full mtDNA genome) [6,20], only haplogroups D and E are pertinent to understanding prehistoric dispersal across the Pacific [8,21,22]. A previous study proposed two independent chicken introductions to the Pacific, with haplogroup E representing the initial introduction and haplogroup D a later one [22]. However, a growing body of evidence from ancient and modern DNA studies refutes this finding, indicating that haplogroup D is probably the only authentic lineage prehistorically translocated into the Pacific [8,23]. All archaeological and most modern chickens in Polynesia belong to a restricted set of D haplotypes defined by a set of four diagnostic mtDNA CR single-nucleotide polymorphisms (SNPs), termed ‘Polynesian D’ [8]. Haplotypes containing this set of SNPs are believed to represent the founding chicken mtDNA lineages dispersed by humans across Remote Oceania. To date, these haplotypes have only been found in one location outside the Pacific, the Philippines [8]. This suggests a homeland for the founding mtDNA lineages, consistent with other lines of evidence regarding early Polynesian origins [24]. Although studies examining the genetic relationships between village chickens from ISEA [25–28] and the Pacific [8,22,23,29] have been performed, no reports have been published on chickens from the Sulu archipelago.

The Sulu archipelago is a chain of islands in the southern Philippines, extending southwest from the Zamboanga peninsula towards Sabah, Malaysia. The archipelago forms a strategic maritime corridor between the Sulu and Celebes Seas, historically linking the Philippines with Borneo and broader ISEA. The archipelago is politically divided into the provinces of Basilan, Sulu, and Tawi-Tawi and is home to various ethnolinguistic groups, including the Tausug, Sama, and Yakan peoples, including the Sama Dilaut or Bajau [30,31]. These communities have long-standing maritime traditions and maintain a mixed economy of fishing, agriculture, and small-scale livestock rearing, including village chickens. Sulu archipelago’s relative geographic isolation and cultural distinctiveness, combined with its historical role as a hub for trade, migration, and cultural exchange, provide a unique context for investigating the genetic diversity and regional affinities of translocated domesticated animals, particularly chickens, because it is entangled and figures prominently in the lifeways of the peoples living in the Sulu archipelago [32–38]. Furthermore, the archipelago may have served as a conduit for prehistoric movement to and from the southern Philippines and Borneo [30,39].

The genetics of village chickens from the Sulu archipelago are worth studying since the history, culture, and language of its peoples demonstrate their dependence on chickens, mainly for food and companionship, as well as the dependence of chickens on the peoples for their propagation and survival [40]. This can be attested by the involvement of chicken-based dishes in rituals and ceremonies [34–38], as well as the depiction of chickens in the visual and performing arts [32,33,41]. This meaningful connection between humans and chickens can be referred to as ‘entanglement’ [42]. This entanglement is also evident among the people in Borneo, some of whom are related to the people in the Sulu archipelago [30,39] and broader Indonesia, through their chicken-based dishes [43], rituals [44–48], and the arts [49,50]. In ISEA, in general, the chicken is a very symbolic animal in cockfighting practises [51,52] and cosmology [53]. Additionally, it is postulated that the cultural value of chickens may have predicated its initial domestication and dispersal more than as a source of food [54].

This study aims to examine the genetic diversity of village chickens from the Sulu archipelago by analysing the mitochondrial DNA control region (mtDNA CR) of chickens from the region. To provide a framework for examining the genetic history of chickens in the Sulu archipelago, the generated mtDNA CR sequences were analysed together with available and comparable datasets from the rest of the Philippines and adjacent regions of Indonesia and the Pacific. Here, we present a close genetic association of village chickens from the Sulu archipelago with Borneo more than the rest of the Philippines. This is probably due to the close cultural and linguistic affinities between these regions. Furthermore, the ancient Polynesian D motif was not observed in chickens from the Sulu archipelago.

## 2. Material and Methods

### 2.1. Regulatory and ethics compliance

This study forms part of a broader interdisciplinary research programme aimed at understanding the genetic history of the peoples in Sulu archipelago and Zamboanga. The research programme and its component projects were reviewed by the Philippine Commission on Higher Education, the National Commission on Culture and the Arts, the Max Planck Institute for Evolutionary Anthropology, and the Natural Sciences Research Institute of the University of the Philippines Diliman. Ethical clearance was provided by the University of the Philippines Manila Research Ethics Board (UPMREB 2018−453−01) and the Ethics Council of the Max Planck Society (application no.: 2021−22). As samples were collected from Philippine Indigenous cultural communities or Indigenous peoples, clearances were also obtained from the Philippine National Commission on Indigenous Peoples (Region IX) and the Office for Southern Cultural Communities in Tawi-Tawi and Sulu. Consent was also obtained from the owners of the chickens before sampling. Furthermore, community consultation and subsequent validation, which involved returning to the communities to present the study results, were conducted as part of the study’s ethical commitment to respect local knowledge and ensure culturally appropriate representation of the results.

### 2.2. Study sites and chicken feather collection

**Figure 1.**
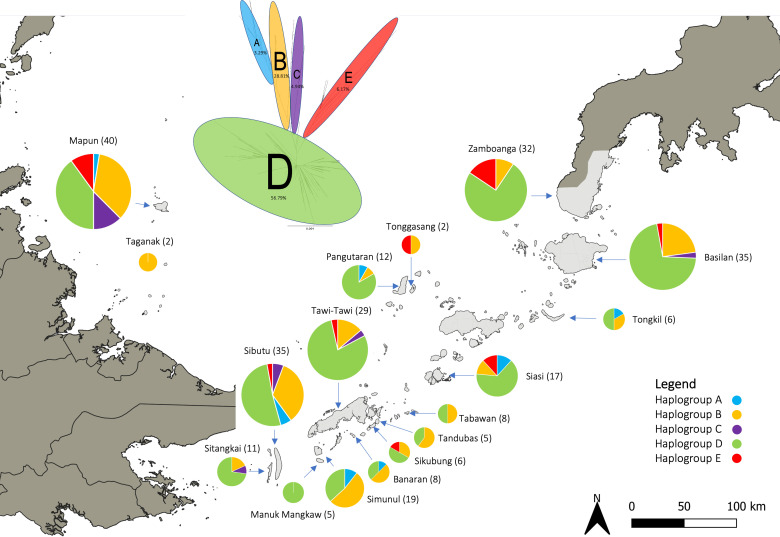
Distribution of chicken mitochondrial DNA control region haplogroups in the Sulu archipelago and Zamboanga peninsula of the Philippines. The inset is a neighbour-joining tree showing the five mitochondrial haplogroups (A, B, C, D, and E) and their relative frequencies in the region.

Body feathers were collected from 254 village chickens across 16 islands in the Sulu archipelago and Zamboanga peninsula (figure 1). The sampling localities and the corresponding number of samples processed for DNA analysis were as follows: Banaran (*n* = 8), Basilan (*n* = 35), Manuk Mangkaw (*n* = 5), Mapun (*n* = 40), Pangutaran (*n* = 12), Siasi (*n* = 17), Sibutu (*n* = 35), Sikubung (*n* = 6), Simunul (*n* = 19), Sitangkai (*n* = 11), Tabawan (*n* = 8), Taganak (*n* = 2), Tandubas (*n* = 5), Tawi-Tawi (*n* = 29), Tonggasang (*n* = 2), Tongkil (*n* = 6), and Zamboanga peninsula (*n* = 14). Flight and tail feathers were avoided due to the thicker calamus, which takes longer to enzymatically digest during DNA extraction. The sampling process was conducted swiftly to minimise disruption to the daily activities of the chicken owners. The collected feather samples, typically 5-10 pieces, were placed in a resealable bag for storage at 4°C until DNA analysis at the University of the Philippines School of Archaeology.

### 2.3. DNA extraction, polymerase chain reaction, sequencing and assembly

Approximately 5 mm of each feather calamus was cut using a clean scalpel. Five feathers were used per chicken depending on the thickness of the calamus. Genomic DNA was extracted using the Qiagen DNeasy Blood and Tissue Kit (Hilden, Germany) following the manufacturer’s instructions, with modifications in the tissue digestion step. Digestion of the cut feather calamus in buffer ATL and Proteinase K was performed overnight at 55°C with the addition of 20 nM dithiothreitol (DTT) as the final concentration.

A partial 764-base pair (bp) fragment of the 1231 bp long mtDNA CR was amplified from the extracted DNA using the primer set: 5'-AACTCCCCTACTAAGGTGTACCCCC−3' and 5'TTGACACTGATGCACTTTGGATCG−3' [28]. Each polymerase chain reaction (PCR), with a final volume of 25 µl, contained 1X HiFi buffer (Invitrogen), 250 µM of each dNTP, 2 mM MgSO_4_, 1 mg ml^−1^ RSA, 0.4 µM of each primer, and 0.5 U of Platinum HiFi *Taq* DNA polymerase (Invitrogen). Thermocycling conditions included an initial denaturation and enzyme activation step at 94°C for 1 min, followed by 50 cycles of denaturation at 94°C for 15 s, primer annealing at 55°C for 15 s, extension at 68°C for 30 s, and a final extension at 68°C for 30 s. Amplicons were sent to the Philippine Genome Center of the University of the Philippines for sequencing using the same primer set. Briefly, the amplicons were purified using the Agencourt AMPure XP beads purification protocol (Beckman Coulter). Purified amplicons were sequenced using the Applied Biosystems BigDye® Terminator v. 3.1 technology on an ABI 3730xl Genetic Analyzer (Thermo Fisher Scientific), and primary analysis (base calling) was performed using the Sequencing Analysis Software v. 5.4 (Thermo Fisher Scientific). Sequence chromatograms were assembled, visually inspected, and manually edited using Geneious [55] to obtain consensus sequences.

### 2.4. Phylogeography, population variability and structure

The newly generated chicken mtDNA CR sequences (*n* = 254; GenBank accession numbers PV420124–PV420377) from this study were aligned with published mtDNA sequences (electronic supplementary material, S1) from the Philippines, Indonesia, and the Pacific [26–28,56,57] using the MUSCLE algorithm [58] in [55]. The inclusion of comparable mtDNA CR sequences from regions in proximity to the Sulu archipelago was aimed at determining geographic distribution of mtDNA CR lineages. The aligned sequences were trimmed to match the 764 bp fragment corresponding to base positions 15 596–16 359 of the mtDNA genome using NC_053523.1 (National Center for Biotechnology Information direct submission). Haplogroup assignments for the newly generated mtDNA CR sequences were made by comparing them with sequences of known haplogroup designations following previously published studies [6,8,20,23]. This was done by generating a neighbour-joining tree [59] and by collapsing the sequences into unique haplotypes using FaBox 1.40 [60]. A median-joining (MJ) network was estimated for the newly generated mtDNA CR sequences of chicken samples from the Sulu archipelago and Zamboanga peninsula using PopArt [61] to investigate evolutionary relationships between haplotypes within a population. Given the central role of haplogroup D in ISEA [28,62] and the initial colonisation of the Pacific [8], subsequent analyses were performed only on the haplogroup D sequences.

Measures of genetic diversity (haplotype and nucleotide), population genetic differentiation (pairwise F*_ST_* scores), and population expansion statistics (Tajima’s D and Fu’s F*s*) were calculated for each sampling locality, which mostly corresponds to islands in ISEA and the Pacific, using Arlequin v. 3.5.2.2 [63]. The Paleontological Statistics Software [64] was used to visualise the differentiation between populations and infer genetic structure by performing a principal coordinates analysis (PCoA) using the population pairwise F*_ST_* scores. Additionally, analysis of molecular variance (AMOVA) [65] was performed to further assess genetic structure in the chicken mtDNA CR dataset. AMOVA was done on the overall dataset from ISEA and the Pacific, assuming no groups and with groups between populations found in the Philippines, Indonesia, and the Pacific islands.

## 3. Results

### 3.1. Mitochondrial control region distribution patterns and diversity

The newly generated mtDNA CR sequences (*n* = 254) of village chickens from Sulu archipelago and Zamboanga peninsula were classified into 5 of the 13 known chicken mtDNA haplogroups—Haplogroups A, B, C, D, and E (figure 1, electronic supplementary material, S2). Haplogroup D was the most prevalent in the region, accounting for 56.79%, and was observed across most of the islands of the Sulu archipelago and Zamboanga peninsula. Haplogroup B was the second most abundant at 28.81%, whereas haplogroups A, C, and E were found at much lower frequencies of 3.29%, 4.94%, and 6.17%, respectively.

**Figure 2.**
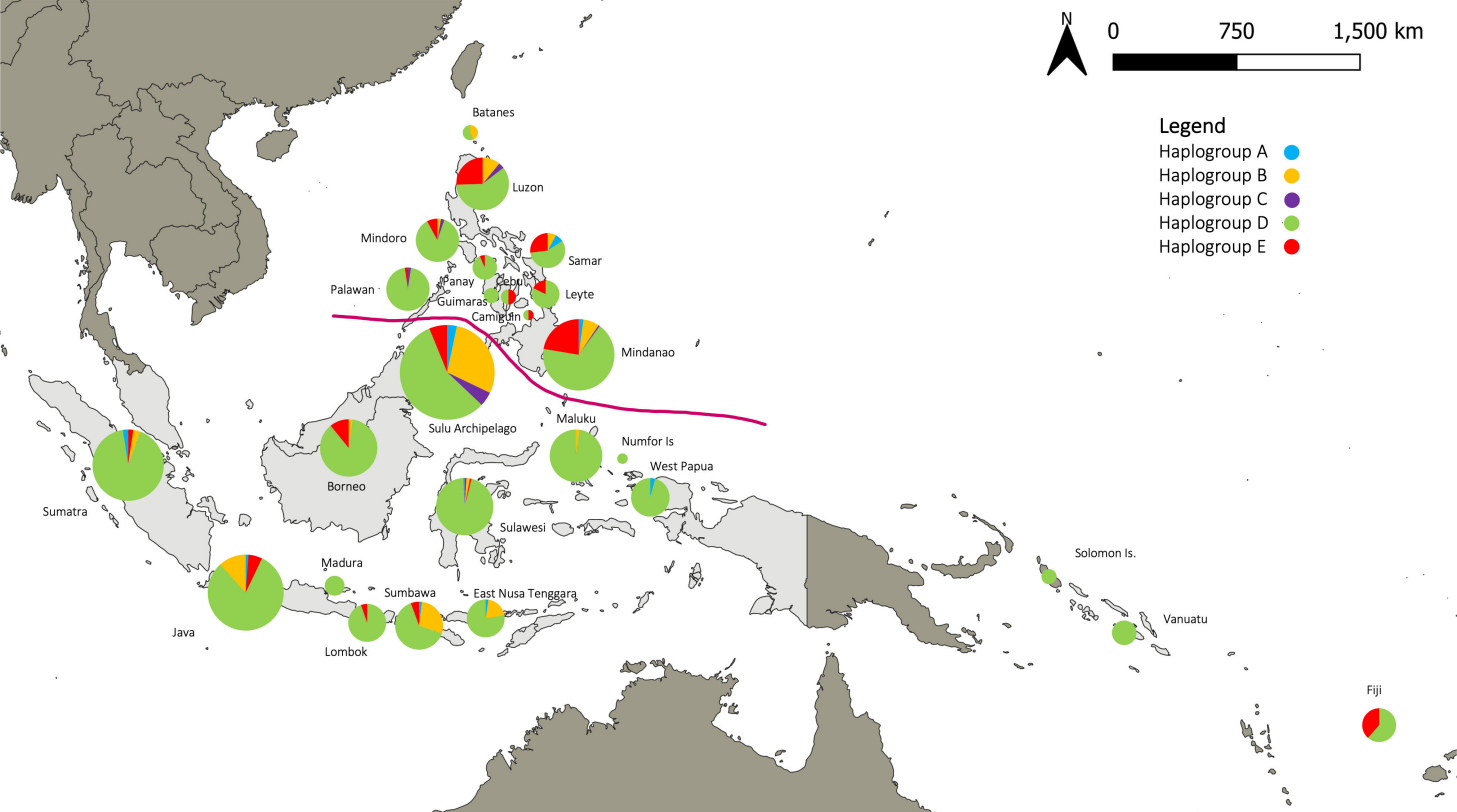
Frequency of chicken mitochondrial haplogroups A, B, C, D, and E in Island Southeast Asia and the Pacific. The line demarcates the area where Polynesian D haplotypes were found in the Philippines.

At a broader regional scale, the dominance of haplogroup D was evident in the Sulu archipelago and in the rest of the Philippines, Indonesia, and the Pacific (figure 2, electronic supplementary material, S3). The maternal genetic structure of chickens in the region appears to be largely driven by haplogroup D. Haplogroup D chickens are the most diverse in the Philippines, with a haplotype diversity of 0.9335, followed by Indonesia at 0.7762, and then the Pacific at 0.1403 (table 1). The Sulu archipelago had a haplotype diversity of 0.9123. Additionally, neutrality tests (Tajima’s D and Fu’s Fs) indicated a significant expansion signal for chicken populations in ISEA and the Pacific belonging to haplogroup D (table 1). This deviation from neutrality supports a model of demographic expansion of haplogroup D from each study region.

**Table 1.** Population genetic summary statistics for mtDNA CR haplogroup D sequences. HD (SD), haplotype diversity (standard deviation); ND (SD), nucleotide diversity (standard deviation); N(h), sample size (number of haplotypes); Pi, mean number of pairwise difference.

genetic diversity				neutrality test		
region/ localities	N (h)	HD (SD)	ND (SD)	Pi	Tajima’s D	Fu’s Fs
**Sulu Archipelago**	**138** (**35**)	**0.9123 (+/-0.0148**)	**0.0785 (+/-0.0468**)	**2.5906**	**−1.6853^a^**	**−25.99944^a^**
Banaran	3 (3)	1.0000 (+/-0.2722)	0.6666 (+/-0.6285)	2.0000	1.0000	−0.6931
Basilan	25 (14)	0.9100 (+/-0.0433)	0.1549 (+/-0.0920)	3.2533	−1.5073^a^	−6.0129^a^
Manuk Mangkaw	5 (2)	0.6000 (+/-0.1753)	0.6000 (+/-0.5310)	1.2000	1.4588	1.6875
Mapun	16 (9)	0.9083 (+/-0.0479)	0.2819 (+/-0.1706)	3.3833	−0.2461	−2.1130
Pangutaran	10 (6)	0.8889 (+/-0.0754)	0.2703 (+/-0.1968)	1.6222	−0.9727	−2.4026
Siasi	11 (4)	0.6909 (+/-0.1276)	0.3151 (+/-0.2193)	1.8909	−0.3230	0.6391
Sibutu	18 (9)	0.8366 (+/-0.0751)	0.1718 (+/-0.1093)	2.4052	−1.5392^a^	−2.9334
Sikubung	3 (3)	1.0000 (+/-0.2722)	0.6666 (+/-0.6285)	2.0000	1.0000	−0.6931
Simunul	7 (5)	0.9048 (+/-0.1033)	0.4217 (+/-0.2868)	2.9523	0.1725	−0.7366
Sitangkai	8 (6)	0.8929 (+/-0.1113)	0.3055 (+/-0.2057)	2.7500	−1.0176	−1.8162
Tabawan	4 (4)	1.0000 (+/-0.1768)	0.5000 (+/-0.4194)	2.0000	−0.7801	−1.8718
Tandubas	2 (2)	1.0000 (+/-0.5000)	1.0000 (+/-1.1180)	4.0000	0.0000	1.3862
Tawi-Tawi	23 (14)	0.9486 (+/-0.0248)	0.1956 (+/-0.1235)	2.3478	−0.9631	−9.0996^a^
Tongkil	3 (3)	1.0000 (+/-0.2722)	0.6666 (+/-0.6285)	2.0000	0.0000	−0.6931
**Philippines**	**297** (**70**)	**0.9335 (+/-0.0067**)	**0.0592(+/-0.0345**)	**2.9049**	**−1.8049^a^**	**−26.0756^a^**
Batanes	4 (1)	—	—	—	—	—
Cebu	2 (2)	1.0000 (+/-0.5000)	1.0000 (+/-1.1180)	4.0000	0.0000	1.3862
Guimaras	4 (3)	0.8333 (+/-0.2224)	0.6250 (+/-0.5031)	2.5000	1.3652	0.4611
Leyte	31 (15)	0.8882 (+/-0.0390)	0.1917 (+/-0.1166)	2.6838	−0.7761	−7.3667^a^
Luzon	30 (12)	0.8897 (+/-0.0353)	0.2290 (+/-0.1411)	2.5195	−0.2979	−4.1368
Mindanao	71 (24)	0.8837 (+/-0.0264)	0.1371 (+/-0.0808)	2.8804	−1.0274	−13.4076^a^
Mindoro	33 (13)	0.8485 (+/-0.0519)	0.1768 (+/-0.1105)	2.2992	−0.9138	−5.4581^a^
Palawan	41 (16)	0.9207 (+/-0.0206)	0.2067 (+/-0.1231)	2.8951	−0.3619	−6.3588^a^
Panay	14 (7)	0.8022 (+/-0.0936)	0.3328 (+/-0.2170)	2.3296	0.2154	−1.6120
Samar	22 (12)	0.9221 (+/-0.0326)	0.1837 (+/-0.1095)	3.3073	−1.2146	−4.1163
Sorsogon	3 (3)	1.0000 (+/-0.2722)	0.6666 (+/-0.6849)	1.3333	0.0000	−1.2164
Zamboanga	24 (15)	0.9312 (+/-0.0384)	0.1677 (+/-0.1020)	2.8514	−1.3396	−8.9091^a^
**Indonesia**	**563** (**86**)	**0.7742 (+/-0.0153**)	**0.0285 (+/-0.0192**)	**1.4254**	**−2.2204^a^**	**−27.7768^a^**
Borneo	40 (12)	0.8282 (+/-0.0473)	0.1806 (+/-0.1181)	1.8064	−0.6908	−5.0148^a^
East Nusa Tengara	35 (12)	0.8336 (+/-0.0505)	0.1915 (+/-0.1308)	1.5327	−0.6190	−6.6010
Java	137 (23)	0.7023 (+/-0.0272)	0.0722 (+/-0.0519)	1.1552	−1.6230^a^	−21.3721
Lombok	18 (8)	0.6993 (+/-0.1171)	0.1220 (+/-0.0937)	1.0980	−20565^a^	−4.0303
Madura	9 (3)	0.5556 (+/-0.1653)	0.3611 (+/-0.3356)	0.7222	−0.0638	−0.2392
Maluku	49 (8)	0.7081 (+/-0.0544)	0.1241 (+/-0.0946)	0.9931	−1.2229	−3.0661
Numfor Is.	2 (1)	—	—	—	—	—
Sulawesi	98 (29)	0.8555 (+/-0.0296)	0.0690 (+/-0.0445)	1.7944	−1.9334^a^	−26.9041
Sumatra	118 (25)	0.7445 (+/-0.0328)	0.0540 (+/-0.0373)	1.3505	−2.0798^a^	−23.5124
Sumbawa	34 (7)	0.7094 (+/-0.0595)	0.1448 (+/-0.1108)	1.0142	−1.1695	−2.4842^a^
West Papua	23 (6)	0.6482(+/-0.0938)	0.1490 (+/-0.1147)	1.0434	−1.4220^a^	−1.9036
**Pacific**	**28** (**3**)	**0.1403 (+/-0.0871**)	**0.0714 (+/-0.1183**)	**0.1428**	**−1.5106^a^**	**−2.2679^a^**
Fiji	16 (2)	0.1250 (+/-0.1064)	0.1250 (+/-0.2262)	0.1250	−1.1622	−0.7001
Solomon	3 (1)	—	—	—	—	—
Vanuatu	9 (2)	0.2222 (+/-0.1662)	0.2222 (+/-0.3267)	0.2222	−1.0882	−0.2634

^a^ statistically significant *p*-values (*p* < 0.05 for Tajima’s D, *p* < 0.02 for Fu’s Fs)

### 3.2. Population structure of haplogroup D

**Figure 3.**
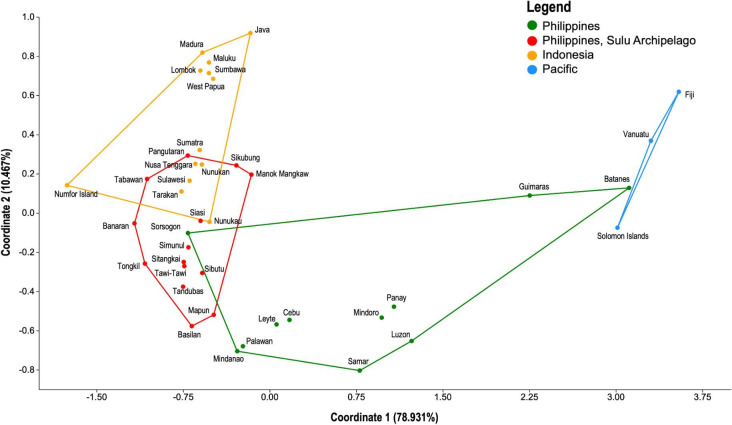
Principal coordinate analysis (PCoA) plot on population pairwise F_ST_ for chicken samples using only haplogroup D. Convex hull colour represents regions used in the study, including the Philippines (green), Sulu archipelago of the Philippines (red), Indonesia (orange), and the Pacific islands (blue).

The PCoA plot of pairwise F_ST_ values (electronic supplementary material, S4) revealed the genetic differentiation among the populations in this study (figure 3). Chickens from the Sulu archipelago showed a closer maternal genetic relationship with samples from the Indonesian islands as compared with the rest of the Philippines. In particular, chickens from the Sulu archipelago were closest to those from Borneo (i.e., Nunukan, Nunukau, and Tarakan), followed by Sulawesi and Sumatra. The major axes of variation in PC 1 (78.931%) revealed that, in addition to the Sulu archipelago chickens, only chickens from Sorsogon, Palawan, and Mindanao appear to be genetically affiliated with Indonesian chickens. In contrast, Pacific chickens are generally closer to Philippines samples, particularly those from Batanes and Guimaras Island. The PCoA plot also shows that Philippine populations occupy a larger space, suggesting a higher genetic diversity compared with the other regions studied (table 1). Furthermore, only a small subset of diversity from the Philippines was shared with the Pacific chickens.

AMOVA showed that a high proportion of genetic variation was found within populations (table 2). The highest variance component for haplogroup D was observed in the population hypothesis based on Philippine/Pacific versus Sulu/Indonesia groupings at 22.12%. The genetic structure was also observed at 21.24% if the haplogroup D chicken populations were grouped into their corresponding regions (Philippines, Sulu archipelago, Indonesia, and the Pacific). However, the among-group variance component drastically decreased when the Sulu archipelago samples were grouped with the Philippines and the Pacific (14.11%). There was a generally high variation observed within populations and low variation among populations in the AMOVA test. This probably indicates that the regions under study experienced frequent gene flow.

**Table 2.** Population genetic structure estimated from the analysis of molecular variance (AMOVA) based on the chicken haplogroup D mtDNA control region sequences from the Sulu archipelago, Philippines, Indonesia, and the Pacific.

				variance components (%)^a^		
group	n	number of populations	number of groups	among groups	among populations within groups	within populations
no groups	1007	41	1	**…**	**20.84**	**79.16**
regions	1007	41	4	**21.24**	**5.78**	**72.98**
Philippines/Pacific versus Sulu/Indonesia	1007	41	2	**22.12**	**8.31**	**69.57**
Philippines/Pacific/Sulu versus Indonesia	1007	41	2	**14.11**	**12.01**	**73.88**
Philippines versus Pacific/Sulu/Indonesia	1007	41	2	**14.83**	**12.73**	**72.44**

^a^ Statistically significant *p*-values (*p* < 0.05)

**Figure 4.**
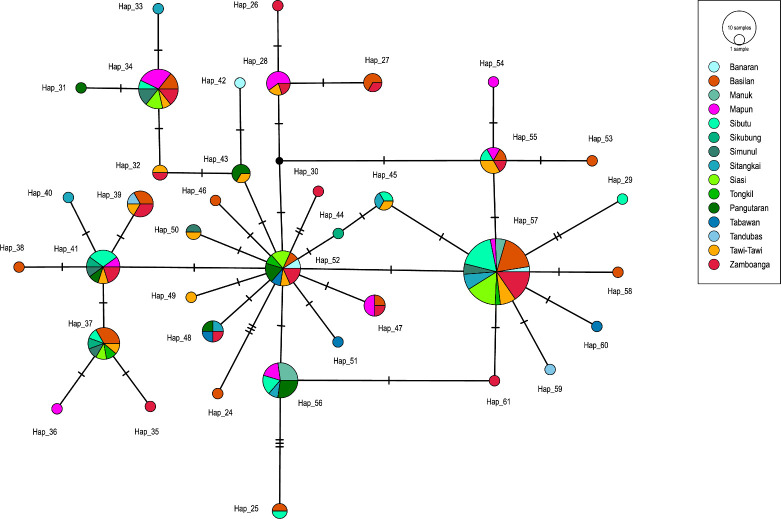
Median-joining network generated using the 764 bp mitochondrial CR sequences classified as haplogroup D from the Sulu archipelago and Zamboanga peninsula. Pie charts are coloured by island, pie size indicates frequency and hashes correspond to the number of mutations between haplotypes. The black dot is an intermediate haplotype that is inferred but not directly observed in the data.

The MJ network of haplogroup D chicken sequences (*n* = 138) from the Sulu archipelago and the Zamboanga peninsula revealed 37 distinct haplotypes (figure 4). Phylogenetic structure is not observable within the Sulu archipelago of the Philippines. The major D haplotypes are widely shared across several islands in the region, with haplotype 57 being the most common. A starburst configuration of the network suggests a potential history of expansion in the region. The complete set of four diagnostic ancestral Polynesian SNP motif composed of A to G at base 281, C to T at base 296, T to C at base 306 and A to G at base 342 [8] was not observed in any of the D haplotypes from the Sulu archipelago. However, this has been observed elsewhere in the Philippines (electronic supplementary material, S3). Haplotype D chickens, containing the Polynesian motif, are the most frequently observed haplotypes in both archaeological and modern chickens in Polynesia [8]. In the study dataset, a total of 11 Polynesian D haplotypes are observed: nine are found in the Philippines and two are from the Pacific (electronic supplementary material, S3). Haplotype 61 is the most frequent, with 45 sequences, and is the only one shared between the Philippines and the Pacific. In the Philippines, the Polynesian D haplotypes are commonly found in Luzon Island, followed by Visayas, and are infrequently observed in Mindanao. However, the Polynesian D motif has yet to be detected in the Sulu archipelago and Indonesia.

## 4. Discussion

Village chickens from the Sulu archipelago predominantly belonged to mtDNA CR haplogroup D (56.79%), followed by haplogroup B (28.81%) (figure 1). Haplogroup D probably represents the initial lineage, as it is the most frequent and diverse lineage in the ISEA. The chicken mitochondrial haplogroup D is genetically divergent from haplogroup B [20]. Haplogroup B is one of the most widely distributed chicken mtDNA lineages in mainland Asia [6], and its presence in the Sulu archipelago probably reflects subrecent or modern-day translocations of chickens into the region. This hypothesis can be addressed directly through ancient DNA analysis of archaeological chicken bones. However, this is challenging because chicken remains are rare in MSEA [66] as well as in the Philippines [67]. Within haplogroup D, the MJ network analysis showed that the mtDNA CR genetic structure within the Sulu archipelago was not differentiated, suggesting a high level of gene flow between the islands (figure 4). Most of the major D haplotypes were shared across the Sulu archipelago, with no apparent signal for translocation. This probably reflects the cumulative long-term interactions of the different communities within the region, either through trade or migration, over an extended period.

As shown by the genetic evidence, it can be argued that the peoples of Sulu archipelago and chickens, especially with Haplotype D, have ‘entangled relations’, where the social life of the communities is defined and practised through their relationship with their chickens [68]. The people of the Sulu archipelago depend on their chickens for food, companionship and connections to the spiritual world, as well as these chickens depend on them for their propagation, dispersal, and survival. Managing and exploiting chickens involving them in foodways, feasts, ceremonies, and games (cockfighting) granted the people in Sulu archipelago overlapping spaces with the chickens (figure 6A) and regular access influenced their interactions with their chickens [40]. For example, in the Tausug population, chicken *piyanggang* (roasted chicken) is one of the native dishes served during *Maligay Pagtammat*, a tradition that has been maintained through the years to symbolise the completion of Quranic studies by children [37]. *Piyanggang manuk* is also prepared to honour ancestors believed to be the supernatural cause of some illnesses during *pagkaja* or healing ritual, which specifically calls for 11 chickens to be prepared with a prayer chant, killing, processing, coating with the *pamapa* mixture (ground burnt coconut meat, turmeric and ginger), boiling to tenderise, and roasting [36]. Another example from the same group also involves the roasted chicken in the *pagkawin*, which is a marriage ritual [38]. Chickens figure prominently in the culture of the peoples in Sulu archipelago, such as the use of wild red jungle fowl in ceremonies of the Tausug people [34], the killing of chickens in the ritual of the Sama-Bajau people [35], the depiction or pattern of *manuk* or chicken into the traditional *okir* design of the Sama Dilaut [32] or *suluk ukkil* design on the *barung* in the Sulu Sultanate [33], and the *kagis-kagis* dance step depicting a chicken scratching the earth [41]. The belief in the chicken as a sacred animal [69] may reflect Hindu influences integrated into the indigenous belief system of the people of the Sulu archipelago [70,71]. In Hindu mythology, the chicken or rooster is depicted as the *vahana* (vehicle) of deities, such as Bahuchara Mata—the mother goddess revered as creator and guardian of life [69,72].

**Figure 5.**
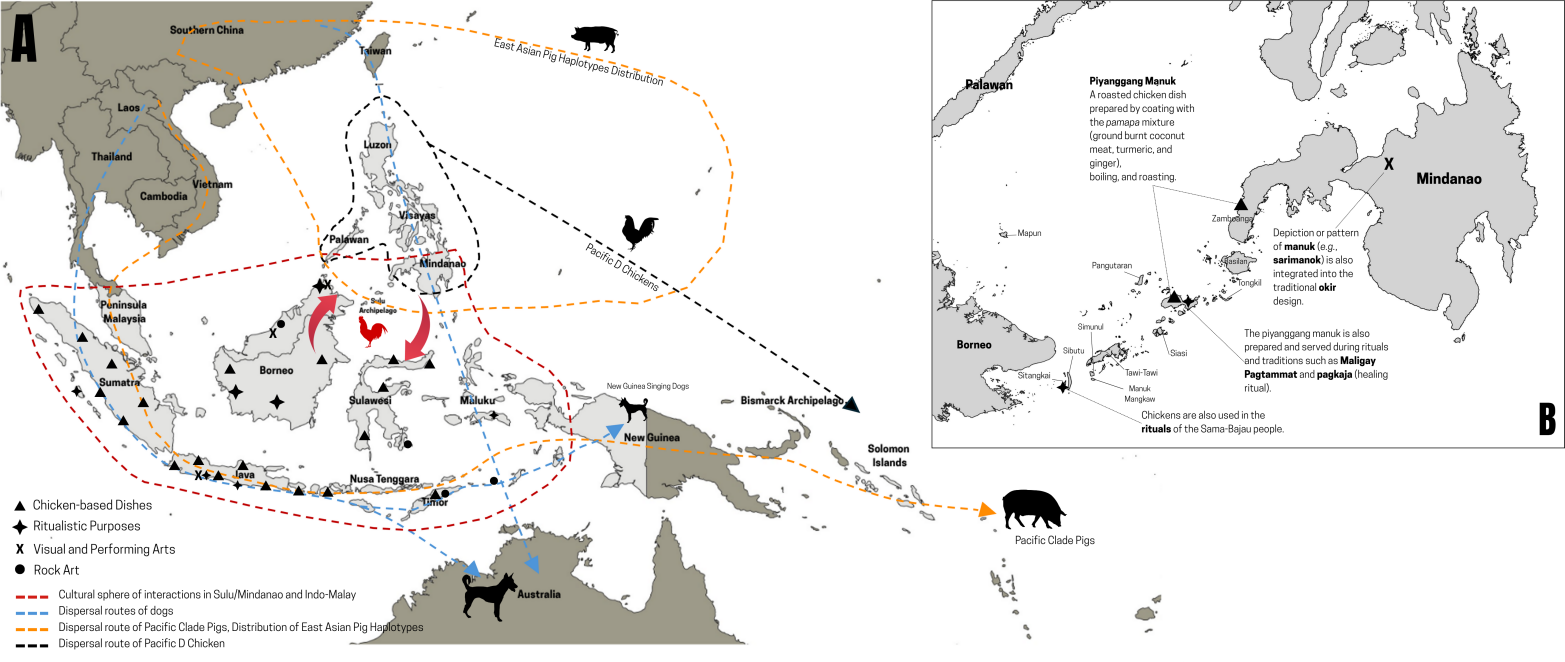
(A) Map of Island Southeast Asia showing the inferred gene flow of key domesticates—chickens, pigs, and dogs—across the region. (B) The map also highlights distribution of shared cultural elements, including chicken-based dishes, ritual practises, visual art motifs (e.g., *okir*), and rock art. Together, these layers illustrate the intertwined movement of animals, people and cultural traditions throughout Island Southeast Asia and into the Pacific.

This human–chicken entanglement [42] can also be demonstrated by the people in Borneo, some of whom are close relatives of the peoples in the Sulu archipelago, especially the Sama [30,39,73]. In Sarawak, Malaysian Borneo, the state’s iconic dish is *ayam pansum*, a chicken cooked in bamboo [43]. It is also called *siok tribuh* by the Bidayuhs in Sarawak. They also have other chicken-based dishes called *nasi sum* (chicken rice) and *saam* (twice-cooked chicken rice in bamboo) [43]. Aside from food, the chicken is also a sacrificial animal in rituals of the Luangan people in Indonesian Borneo [44], Bidayuh people of Sarawak, Malaysian Borneo [47], Bentian people of East Kalimantan [48] and the Iban people of Santubung, Sarawak [46]. The chicken is also sacrificed after a successful augury, as ensured by the prayers, demonstrating the importance of chickens in the religious practise in Borneo [45]. The depiction of jungle chicken in the Painted Cave within the Niah Cave Complex indicates that the chicken has been a significant food source since the Neolithic period in Borneo and ISEA [50]. Chickens are also depicted in the rock art at the Gua Metanduno, Muna Island, Southeast Sulawesi [74]; Wetang Island, Maluku Barat Daya [75]; and Ilé Kéré Kéré, Nino Konis Santana National Park, Muapitine, Timor-Leste [76], the latter depicting two chickens fighting.

When the newly generated mtDNA CR sequences from Sulu archipelago were compared with adjacent regions, a previously undescribed substructure was evident. Within the Philippines, haplogroup D chickens from Sorsogon, Palawan, and Mindanao exhibit a closer genetic affinity to those from the Sulu archipelago than those from other regions (figures 3 and 5b). The genetic link with Mindanao, in particular, may reflect long-standing Indian-Malay cultural influences that pre-date the broader wave of Islamisation in the area [70,71]. Village chickens from the Sulu archipelago show greater genetic affinity with chickens from Borneo and Indonesia, in general, than the rest of the Philippines (figure 3). This is not entirely surprising, as communities in the Sulu archipelago share cultural and linguistic components with Indonesia [31]. Further demonstrating the interactions of humans with chickens in wider Indonesia, the red junglefowl (*Gallus gallus*) is depicted in one of the panels in the Lalitavistara relief of Borobudur Temple [49]. Aside from being the most accessible protein source for food and as a ritual ingredient, chickens also serve as time markers because they crow at the same time every morning [49]. Although chickens or *manuk* are not forbidden as food in any religion [49], they cannot be killed and eaten by the Huaulu people of eastern Indonesia because they are considered as ‘quasi-human’ beings [77]. Chicken is the most common meat in Indonesian cuisine [78], one of the sources of offal for diverse offal-based dishes in Indonesia [79], one of the domesticated animals traditionally reserved for feasts [80], essential to rituals alongside with pigs [81] and one of the symbolic animals [82]. Domestic animals used for sacrifice, such as chickens, serve as carriers of prayers to the deities as messengers, where the response if the request was granted can be seen in the entrails of these animals [83]. The Naga people of Tasikmalaya, West Java have a folk classification of village chickens, demonstrating their recognition of variation of chickens [84]. Having 31 breeds of native chickens in Indonesia demonstrates genetic richness from decadal development of native chicken farming [85]. It is even claimed that Indonesia was one of the centres of domestication of chickens [54].

Across the wider ISEA, the entanglement of humans with chickens can be demonstrated by cockfighting practises with associated meanings [51,52]. The practise of cockfighting symbolises masculinity [51], like what the chicken symbolises in the *suluk ukkil* in the Sulu Sultanate [33]. From the perspective of multispecies ethnography involving the Kampung Laut people in Segara Anakan, Indonesia, cockfighting is an interspecies collaboration between humans and non-human species (chicken), where the fighting roosters possess their agency to make decisions and impact humans [52]. The non-food importance of chickens, based on examples from history, culture, and language, may have been the catalyst for their initial domestication and dispersal, rather than their role as a source of food [54]. Also, the constellation known as the bird or *manuk*, featuring the stars Sirius and Canopus, is visible and part of cosmology across the ISEA [53]. However, it remains challenging to speculate on the origin and initial direction of this chicken translocation without ancient DNA data from the region despite supporting evidence from culture, history, and language.

Regarding the source population for Pacific chickens, the Philippines remains the likely origin of chickens transported into Near and Remote Oceania (figure 5A) [8,57,62], which aligns with linguistic and archaeological models for the Austronesian expansion [2]. This process probably began with naturally isolated populations within the Philippines, followed by subsequent founder effects and *in situ* evolution as chickens were transported across the Pacific. The localised collection and transport of chickens would have involved only a few closely related lineages. Austronesian-speaking migrants probably selected a small subset of available chickens (specifically from the broader haplogroup D diversity) from the Philippine parent population. The geographic distribution of the Polynesian D haplotypes, particularly their absence in the Sulu archipelago and Indonesia, suggests that these regions did not contribute to the initial maternal lineage of Pacific chickens east of Near Oceania. The lack of Polynesian D haplotypes in eastern Indonesia also suggests a direct route of human-mediated transfer of chickens from the Philippines to Near Oceania. Furthermore, linguistic evidence supports this hypothesis that the origin of Pacific chickens lies in the Philippines, rather than Taiwan (the hypothesised linguistic homeland), as there are no known Proto-Austronesian (Formosan) terms for chickens [1]. The Austronesian term for chicken first appears in the Proto Malayo-Polynesian language, which developed in the Philippines and is ancestral to all other Austronesian languages in the Pacific [86].

The characterisation of the chicken mtDNA CR dataset from Indonesia and the Philippines demonstrates that the ISEA, in general, is a centre for haplogroup D diversification. The ubiquity of haplogroup D chickens in ISEA also suggests a long-standing presence in the region. Unfortunately, archaeological chicken remains have yet to be documented in ISEA, making it challenging to investigate the temporal depth of haplogroup D in this region. Nevertheless, the presence of Polynesian D haplotypes at the Teouma site in Vanuatu dating before *ca* 3000 yBP [19] suggests they must have been transported through the ISEA before this date.

## 5. Conclusion

The close genetic association of village chickens from the Sulu archipelago with chickens from Indonesia, particularly Borneo, is probably due to the strong entanglement of the diverse cultures found in the region. The Sulu archipelago and Indonesia share a complex, deeply intertwined history shaped by cultural, linguistic, economic, and political connections that stem from their proximity and shared Austronesian roots. However, the close genetic affinity of chickens from the Sulu archipelago with those chickens further south suggests a complex translocation history—one probably shaped by trade networks that brought Indian-Malay influence to the southern Philippines. These interactions, particularly in regions, such as Mindanao and the Sulu archipelago, introduced Indian religious, artistic, and linguistic elements, along with the possible translocation of chickens. The lack of genetic structure in the key chicken mtDNA CR lineage, haplogroup D, may be due to the Sulu archipelago’s role as an important maritime crossroads. Its strategic position in the ISEA made it an exchange centre for ideas, goods, and people. Furthermore, the movement of people, particularly maritime nomadic groups like the Sama Dilaut, contributed to the formation of transnational communities spanning both the Sulu archipelago and Indonesia. With the newly generated chicken mtDNA data available from the Sulu archipelago of the Philippines, we now have some insights into the southernmost distribution of chickens with the ancestral Polynesian D motif on the southern part of the Philippines. Its absence in the Sulu archipelago could potentially be attributed to past population replacements in the region or unsuccessful initial establishment. Alternatively, chickens with the ancestral Polynesian D motif might not have arrived in the region at any point in the past. This hypothesis can be directly tested through ancient DNA analysis of archaeological chickens excavated from the Sulu archipelago.

## Data Availability

All mtDNA control region sequences generated in this study are available in GenBank under accession numbers (PV420124 - PV420377). Supplementary material is available online [87].
